# Metabolomic Signatures of Longevity in Bivalves Reveal Reorganization of TCA Cycle and Amino Acid Metabolism

**DOI:** 10.1111/acel.70772

**Published:** 2026-10-25

**Authors:** Pauline Bertrand, Clément J. L. Chevret, Enrique Rodríguez, Hélène Lemieux, Pierre U. Blier

**Affiliations:** ^1^ Department of Biology Université du Québec à Rimouski Rimouski Quebec Canada; ^2^ Department of Medicine University of Alberta Edmonton Alberta Canada; ^3^ Research Department of Genetics, Evolution and Environment University College London London UK; ^4^ Faculté Saint‐Jean, University of Alberta Edmonton Alberta Canada

**Keywords:** aging, amino acids, hypoxia, longevity, metabolomics, mitochondria, Mollusca/Bivalvia, tricarboxylic acid cycle

## Abstract

Mitochondria are central regulators of cellular metabolism, integrating energy production, redox balance, and signaling pathways that shape aging trajectories. If mitochondrial organization contributes to lifespan divergence, exceptionally long‐lived species should exhibit distinctive metabolic signatures reflecting adaptations promoting long‐term cellular maintenance. Comparative approaches across species with extreme lifespan variation provide a powerful framework for identifying potential metabolic strategies underlying longevity. We conducted targeted metabolomic profiling of five marine bivalve species spanning a lifespan gradient from approximately 30 to more than 500 years, including the exceptionally long‐lived ocean quahog, *Arctica islandica* . Fifty metabolites were quantified in gill and mantle tissue to determine whether lifespan variation is associated with coordinated changes in metabolic organization. Lifespan emerged as a dominant axis of metabolomic variation across species. Long‐lived bivalves exhibited a distinct metabolic phenotype characterized by a global reduction in free amino acid pools and coordinated changes in metabolites associated with mitochondrial metabolism. Longevity was associated with increased succinate‐to‐fumarate ratios, reduced fumarate and malate concentrations, and reduced engagement of pyruvate‐derived anaerobic pathways under basal conditions. Although these patterns varied in magnitude across tissues, they converged on a common longevity‐associated metabolic signature. Together, these patterns suggest that exceptional longevity is associated with a distinct organization of mitochondrial‐related metabolism and a more selective allocation of metabolic resources, rather than a generalized suppression of metabolism. Our findings identify metabolomic signatures associated with exceptional longevity across the bivalve species studied and support the hypothesis that long lifespan is characterized by a distinctive metabolic architecture favoring long‐term cellular maintenance.

## Graphical abstract

Targeted metabolomics of gill and mantle tissues from five marine bivalve species spanning lifespans from approximately 30 to over 500 years revealed longevity‐associated metabolic signatures. Longer‐lived species exhibited reduced free amino acid pools, lactate, pyruvate‐related ratios, fumarate and malate, alongside increased succinate‐to‐fumarate ratio, suggesting a reorganization of intermediary metabolism.

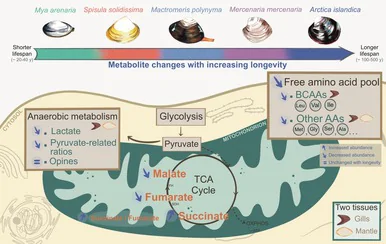

## 1 Introduction

Mitochondria, far beyond their classical role as cellular powerhouses, serve as dynamic integrators of metabolic and signaling processes central to aging trajectories and lifespan regulation (Blier et al. 2017; Akbari et al. 2019). They contribute to cellular homeostasis through redox‐dependent signaling pathways, notably via reactive oxygen species (ROS) and through ion‐dependent signaling, particularly Ca^2+^ handling (Holmström and Finkel 2014; Nicholls 2005). In parallel, mitochondria rely on an intrinsic quality‐control network, including fusion, fission, and mitophagy, to preserve organelle integrity and functional competence over the lifespan (Westermann 2012; Pickles et al. 2018). By coordinating metabolic regulation with stress‐response and quality‐control pathways, mitochondria exert a profound influence on cell fate decisions, shaping processes such as inflammation, adaptive stress signaling, and apoptosis (Murphy and O'Neill 2024; Adebayo et al. 2021). Consequently, mitochondrial function lies at the core of many conserved mechanisms of aging and is increasingly recognized as a key contributor to age‐related and degenerative diseases (Harrington et al. 2023; Sun et al. 2016; Barja 2019).

Comparative approaches have been particularly powerful for identifying mitochondrial features linked to longevity, and among these, bivalves stand out as exceptional models (Blier et al. 2017; Munro et al. 2023). Some species exhibit striking differences in lifespans while sharing relatively conserved body plans and ecological niches. Most notably, the ocean quahog *Arctica islandica* is the longest‐lived non‐colonial metazoan known, with a maximum reported longevity exceeding five centuries (Butler et al. 2013). This extraordinary lifespan has been associated with distinctive mitochondrial functional and structural traits, including remarkable metabolic plasticity that enables the maintenance of efficient mitochondrial function under extreme stressors such as prolonged anoxia, repeated reoxygenation cycles, and severe chemical oxidative challenges (Steffen et al. 2021; Sokolova et al. 2019). Across bivalve species, components of oxidative phosphorylation (OXPHOS) vary markedly, with long‐lived taxa exhibiting reduced relative catalytic capacity and lower control strength of electron flux through both the NADH‐ and the succinate‐linked pathways, mediated by Complex I (CI) and Complex II (CII) of the electron transport system (ETS), respectively (Munro et al. 2013; Rodríguez et al. 2021). In addition, long‐lived bivalves display elaborated organization of ETS supercomplexes (Rodríguez et al. 2022), a feature thought to enhance electron channeling while limiting electron leakage and ROS production.

Importantly, convergent mitochondrial signatures have emerged across diverse taxa, suggesting that modulation of mitochondrial electron flux is a conserved feature of lifespan. In the seed beetle ( *Acanthoscelides obtectus* ), for example, lines selected for extended longevity display a reduced NADH‐pathway capacity (Mast et al. 2024). Similarly, in honeybees ( *Apis mellifera* ), long‐lived queens exhibit lower NADH‐pathway capacity in the head and the abdomen than short‐lived workers, despite sharing a similar genome (Chevret et al. 2026). Experimental manipulations of mitochondrial function further support a causal relationship between OXPHOS capacity and longevity: mild, transient pharmacological inhibition of CI extends lifespan in several model organisms (Hirose et al. 2019; Lee et al. 2003), whereas stronger or sustained inhibition shortens lifespan (Camus et al. 2023). Together, these findings suggest that the relationship between mitochondrial respiratory capacity and longevity is not linear, with moderate reductions in electron flux potentially favoring longevity while more severe mitochondrial impairment becomes detrimental.

Beyond mitochondrial respiratory function, increasing evidence indicates that mitochondrial metabolic plasticity is tightly coupled to redox balance and metabolite availability (Borkum 2023; Hu et al. 2020). Across taxa, metabolites have emerged as key regulators of cellular physiology and as central coordinators of longevity‐related responses (Spinelli and Haigis 2018; Sebastiani et al. 2024). Metabolites closely linked to mitochondrial function not only sustain energy demand but also act as signaling cues regulating stress‐response pathways, nuclear gene expression, epigenetic programs, and antioxidant defenses (Frezza 2017; Sciacovelli et al. 2016). Through these mechanisms, mitochondrial metabolic rewiring associated with species‐specific functional divergence may exert long‐lasting effects on proteins and gene regulation, ultimately shaping aging trajectories and lifespan outcomes (Martínez‐Reyes and Chandel 2020).

This growing body of evidence raises a fundamental question: do exceptionally long‐lived species possess a distinct metabolic architecture that could reflect key physiological adaptations for longevity? Although interspecific divergence in mitochondrial structure and regulation, particularly in bivalves, is well established, how these differences manifest at the systemic metabolomic level remains largely unexplored. To address this gap, we compared five bivalve species spanning a wide range of maximum reported longevity (MRL), including the longest‐lived metazoan *Arctica islandica* (507 years), alongside *Mercenaria mercenaria* (106 years), *Mactromeris polynyma* (92 years), *Spisula solidissima* (37 years), and *Mya arenaria* (28 years) (Figure 1A) (Butler et al. 2013; Ridgway et al. 2011; Munro and Blier 2012; MacDonald and Thomas 1980; Sephton and Bryan 1990). We quantified 50 metabolites in two physiologically distinct tissues: (1) the gills, characterized by high cellular turnover and metabolic activity, and (2) the mantle, a more quiescent tissue with lower metabolic demand. Our objective was to determine whether interspecific variation in lifespan is associated with distinct metabolomic signatures and whether these patterns are consistent with previously identified differences in OXPHOS function and ROS production across the lifespan gradient in this comparative bivalve model. By examining metabolomic profiles across species rather than focusing on individual metabolites or isolated pathways, this study aimed to identify metabolic fingerprints associated with longevity and generate new hypotheses regarding the metabolic adaptations that may contribute to divergent aging trajectories.

**FIGURE 1:**
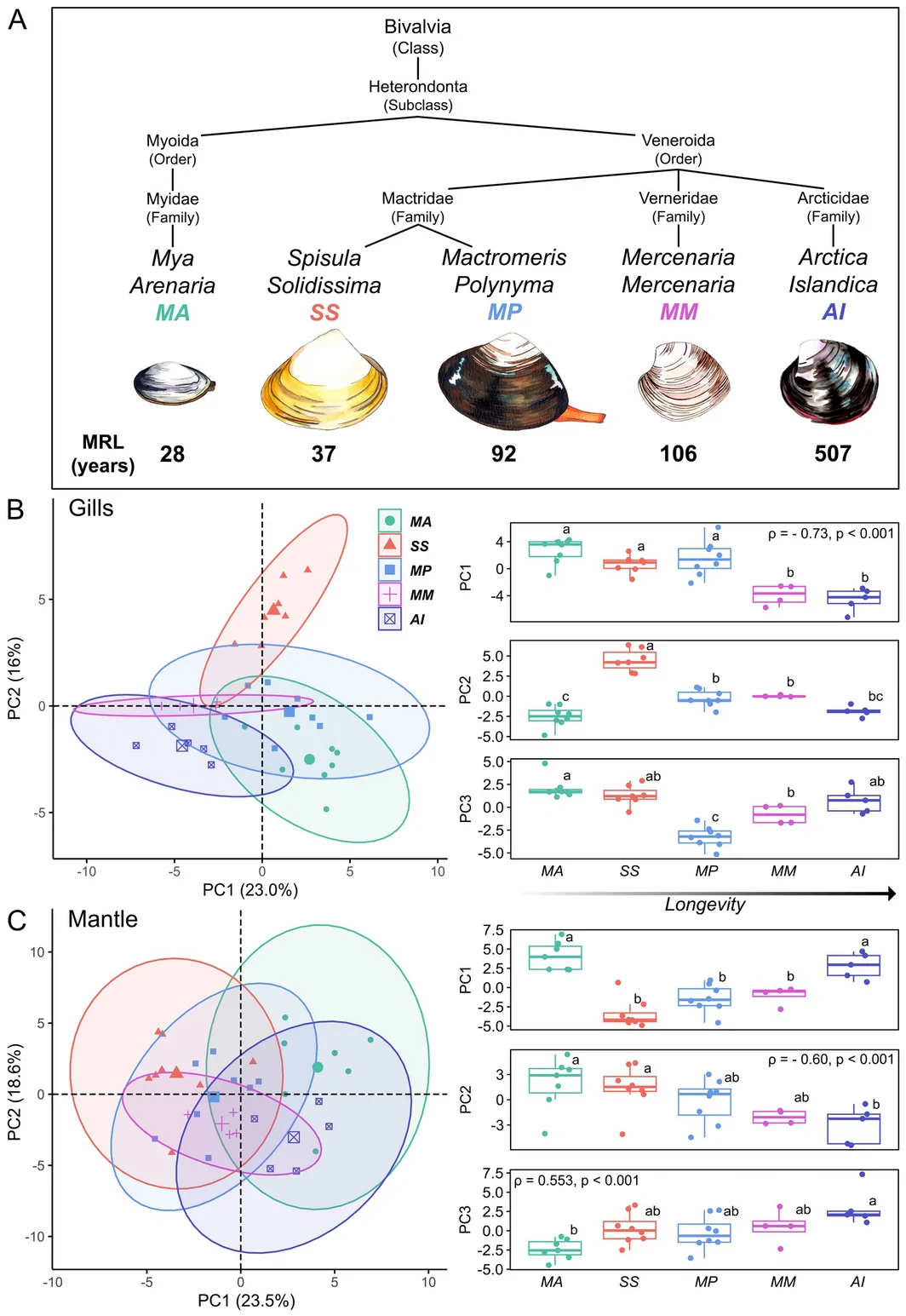
(A) Taxonomy and maximum reported longevity (MRL) of the five bivalve species included in the longevity model. (B, C) Principal component analysis (PCA) of metabolomic profiles in gill (B) and mantle (C) tissues across five bivalve species with contrasting maximum lifespan reported. Biplots show the first two principal components, with points representing individuals and 95% confidence ellipses around species centroids. Data were log_10_‐transformed and autoscaled prior to analysis. Boxplots on the right represent the distribution of sample scores along each principal component; species are listed in order of increasing maximum lifespan. Letters indicate significant differences among species (*p* < 0.05). Spearman's *ρ* values are shown when correlations with the maximum reported longevity (MRL) were statistically significant. *M. arenaria* (MA), *S. solidissima* (SS), *M. polynyma* (MP), *M. mercenaria* (MM), *A. islandica* (AI).

## 2 Materials and Methods

### 2.1 Bivalve Collection

Marine bivalves were collected from multiple sites in eastern Canada. *M. arenaria* were harvested at low tide in the St. Lawrence Estuary near Baie‐Comeau (Quebec, Canada), while *M. polynyma* were obtained offshore in the same estuary by commercial fishers. *A. islandica* were collected by divers near the Magdalen Islands (Quebec, Canada), whereas *S. solidissima* and *M. mercenaria* were collected along the Atlantic coast of New Brunswick (Canada). All individuals were maintained for at least 6 months prior to experimentation at the ISMER marine research station in Pointe‐au‐Père (QC, Canada) in open‐circuit tanks containing sand to allow natural burrowing behavior. Water temperature followed seasonal conditions (12°C in summer to 4°C in winter) and was stabilized between 8°C and 10°C for at least 2 months before sampling, within the thermal comfort zone for all species (Munro et al. 2013). Bivalves were fed a live microalgal mixture (*Nannochloropsis* sp., *Isochrysis galbana*, *Pavlova lutheri* ; 12%, 44% and 44% by cell number, respectively) at a ration equivalent to 1% of total body mass (Munro and Blier 2012). All individuals exceeded the reported size at sexual maturity for their respective species and can therefore be considered mature adults (see Supplementary Methods, Table M1). Gill and mantle tissues were dissected on ice, incubated at 10°C for 10 min in a buffer containing 50 mM NaCl, 400 mM sucrose, 6 mM EGTA, 3 mM EDTA and 70 mM HEPES (pH 7.6) to approximate resting physiological conditions, then flash‐frozen in liquid nitrogen and stored at −80°C. Sample sizes for analyses were as follows: *A. islandica* (*n* = 5), *M. mercenaria* (*n* = 4), *M. polynyma* (*n* = 8), *S. solidissima* (*n* = 8), and *M. arenaria* (*n* = 7), with the same individuals used for both gill and mantle analyses.

### 2.2 Targeted Metabolite Quantification

A total of 50 key metabolites were quantified in gill and mantle tissues of five species with contrasting lifespans. The targeted panel included amino acids (protein metabolism), intermediates of major bioenergetic pathways (TCA cycle, glycolysis), nucleotides (mono‐, di‐, and triphosphates), and metabolites associated with anaerobic metabolism (e.g., lactate, pyruvate, alanine, serine, octopine). Metabolite extraction followed an Agilent‐based protocol (Kennedy and Bivens 2017) adapted by IsoBioKem. Briefly, freeze‐dried samples were weighed (~2 mg) and homogenized three times for 10 s at 4000 rpm using a Precellys 24 homogenizer (Bertin Corporation). Extraction solvent (TFE: LC–MS‐grade water, 1:1, v/v); (TFE: 2,2,2‐trifluoroethanol) was added, followed by vortexing (30 s) and centrifugation (4750 *g*, 5 min). Supernatants from each extraction cycle (750 μL, 750 μL, and 1 mL) were pooled in 4 mL acetonitrile (ACN), vortexed (30 s), and centrifuged (3040 *g*, 5 min). Extracts were evaporated overnight in a SpeedVac concentrator (Savant SPD2010, Thermo Scientific). Dried samples were reconstituted in 250 μL ACN/LC–MS‐grade water (80/20, v/v) and transferred to amber HPLC vials with inserts (Wheaton, NJ, USA). Internal standards were added for positive (valine‐d_8_, 0.1 μgmL^−1^) and negative (pyruvate‐d_3_, 0.1 μgmL^−1^) ionization modes.

Ten microliters of each extract were injected into an Agilent 1260 Infinity II HPLC system coupled to a 6420B Triple Quadrupole mass spectrometer (Agilent Technologies, Santa Clara, CA, USA). Chromatographic separation was performed on an InfinityLab Poroshell 120 HILIC‐Z column (2.7 μm, 10 × 2.1 mm). Two acquisition modes, each with distinct mobile phases and chromatographic conditions, were used to detect all 50 metabolites (see Supplementary Methods for chromatographic and mass spectrometry parameters). Data were processed using MassHunter QQQ quantitative (Quant‐my‐Way) software (Agilent Technologies). Metabolites were quantified using calibration curves with internal standards. Retention‐time windows were verified to account for minor shifts during extraction or loading, and peak areas were normalized to the internal standard signal (MES) for each sample. Metabolite concentrations were expressed as ng.mg^−1^ of dry tissue mass.

### 2.3 Citrate Synthase and Protein Assays

Citrate synthase (CS) activity and protein content were measured from frozen tissue aliquots collected from the same individuals used for metabolomic analyses. CS activity was measured in duplicate at 15°C using a thermostated UV/VIS spectrophotometer (Ultrospec 2100 Pro) by monitoring the reduction of 5,5′‐dithiobis‐(2‐nitrobenzoic acid) (DTNB) at 412 nm following a modified protocol from Thibault et al. (1997), and CS activity was expressed as U mg^−1^ protein. Protein content was measured in triplicate using the bicinchoninic acid (BCA) assay (Smith et al. 1985) with an EnVision Multilabel Plate Reader (PerkinElmer, Waltham, MA, USA).

### 2.4 Statistical Analyses

The few missing values were imputed using a random forest approach (Kokla et al. 2019). Prior to analyses, metabolite concentrations were log_10_‐transformed and auto‐scaled (mean‐centred and scaled to unit variance) separately for each tissue to ensure normality. All subsequent analyses were performed using this normalized dataset. All statistical analyses were conducted in R version 4.4.2 (R Core Team 2024).

#### 2.4.1 Multivariate and Univariate Analyses

Both multivariate and univariate approaches were used to identify metabolites discriminating among species and to assess whether metabolomic profiles cluster according to species identity and lifespan.

Unsupervised principal component analysis (PCA) was performed separately for each tissue to explore the global data structure and to determine whether species grouped by metabolomic signatures aligned along the longevity gradient. Metabolite contributions to each principal component were evaluated using Pearson correlations (loadings) between metabolite abundances and PC axes. Loadings were ranked by absolute value, and metabolites with a |*r*| ≥ 0.7 were retained as the strongest contributors. PC scores were analyzed using one‐way ANOVA followed by Tukey's HSD test; when assumptions of normality or homoscedasticity were not met, Kruskal–Wallis and Dunn's tests were applied. Associations with MRL and shared maximum longevity (SML), defined as the highest age reported in at least two populations, providing a more conservative longevity estimate (Munro and Blier 2012), were assessed using Spearman's rank correlation. Although only MRL is shown in figures, SML yielded identical correlation patterns.

To complement unsupervised analyses, supervised partial least squares discriminant analysis (PLS‐DA) was performed using MetaboAnalyst 5.0 (accessed in November 2024). PLS‐DA reduces dimensionality while maximizing separation among predefined groups. Model performance was evaluated using 5‐fold cross‐validation (classification accuracy and *Q* ^2^), and robustness was assessed via 100 permutations. Metabolite contributions were assessed using variable importance in projection (VIP) scores; metabolites with VIP > 1 were retained for interpretation.

Global differences in metabolomic profiles were assessed using PERMANOVA on Euclidean distance matrices (Anderson 2014). This analysis tested the combined and individual effects of species and tissues, as well as the influence of body size. When PERMANOVA results were significant, pairwise comparisons were performed to identify interspecific differences in multivariate space. For each metabolite within each tissue, normality and homogeneity of variance were tested using the Shapiro–Wilk and Bartlett's or Levene's tests, respectively. When assumptions were met, one‐way ANOVA followed by Tukey's HSD test was used; otherwise, the Kruskal–Wallis test followed by Dunn's test was applied. A Benjamini–Hochberg false discovery rate (FDR) correction was applied to account for multiple testing, with adjusted *p*‐values provided in Table S4. These univariate analyses were used to validate patterns identified in multivariate and regression‐based analyses. To explore pathway‐level variation, biologically meaningful metabolite ratios were calculated to reflect the architecture and balance within central metabolic pathways, including the TCA cycle, glycolysis, energy cofactors, and amino acid metabolism.

#### 2.4.2 Ordinal Logistic Regression: Proportional Odds Model

To identify metabolites associated with the longevity gradient and evaluate their potential as predictors of maximum lifespan, ordinal logistic regression (OLR) models were fitted for each metabolite with species ranked by MRL, considering a linear formulation:

(1) $$\log\left(\frac{P\;\left(Y\leq j\right)}{P\;\left(Y>j\right)}\right)=\zeta_{j}+\beta_{1}X_{i}$$

Here, *X* represents metabolite abundance and *Y* the ordinal longevity rank (MA < SS < MP < MM < AI). Threshold parameters (ζj) define cutpoints between longevity groups, while coefficient $\beta_{1}$ represents the linear effect of metabolite abundance on the log‐odds of belonging to a higher longevity group (Supplementary Methods Figure M1). Standard errors were estimated from the Fisher information matrix, and *z*‐scores were converted to two‐sided *p*‐values using the normal distribution.

Model assumptions were evaluated, particularly the proportional odds assumption, which requires that predictor effects remain constant across thresholds of the ordinal outcome. This assumption was tested using the Brant test (Brant 1990). For some metabolites, this assumption was rejected (Table S4), likely reflecting large interspecific differences and limited overlap in metabolite concentrations. To address these violations, permutation tests (*n* = 1000) were applied to assess the robustness of coefficient estimates. Permutations were applied to $\beta_{1}$ (slope across the longevity gradient). Empirical *p*‐values were calculated as:

(2) $$p=\frac{\#\left(\left|\beta^{\left(k\right)}\right|\geq \left|\beta_{\mathrm{obs}}\right|\right)+1}{n_{\text{perm}}+1}$$

where $\beta^{\left(k\right)}$ denotes the statistic obtained in permutation *k* and $\beta_{\mathrm{obs}}$ is the observed statistic.

Metabolites were considered important when they exhibited both large effect sizes (*β*_1_ far from zero) and statistical significance (−log_10_ *p* > 1.301). Benjamini–Hochberg‐adjusted *p*‐values (FDR correction) were calculated to account for multiple testing. OLR was used as a complementary approach to assess whether significant interspecific differences followed monotonic associations with lifespan. Given the limited number of ordinal categories and the potential influence of extreme values on the regression signal, associations were considered robust only when supported by both regression‐based results and consistent patterns observed in univariate analyses and post hoc comparisons.

## 3 Results

### 3.1 Global Metabolite Patterns: Longevity Emerges as a Dominant Axis of Metabolic Organization

Multivariate analyses revealed significant effects of species (*p* = 0.001), tissue (*p* = 0.007) and their interaction (*p* = 0.001) on global metabolomic profiles, while body size had no detectable influence (*p* = 0.734) (Table S1). All species exhibited distinct metabolomic signatures in both gill and mantle tissues.

Unsupervised PCA revealed clear interspecific separation. In gills, the first principal component (PC1; 23.0% of total variance) aligned strongly with species' MRL, forming a continuous longevity gradient (Figure 1B). This axis was primarily driven by essential amino acids (AAs), including branched‐chain amino acids (BCAAs), threonine and phenylalanine, as well as the non‐essential AAs serine, glycine and alanine (Table S2). In mantle tissue, longevity‐related variation was distributed along PC2 (18.6%) and PC3 (13.1%, Figure 1C): PC2 was mainly associated with AAs, whereas PC3 was dominated by the TCA cycle intermediates fumarate and malate. Correlations between PCA scores and species' MRL supported these associations (Figure 1B,C). Supervised PLS‐DA confirmed the species‐level separation and the longevity gradient (Figure S1). Metabolites driving PCA variance also ranked among the top discriminants (VIP > 1; Table S3), highlighting a consistent metabolomic signature associated with lifespan. Complete metabolite concentration profiles (Figure S2) showed that the most abundant metabolites were largely shared across tissues and included major AAs (e.g., betaine, alanine and glycine) as well as the TCA cycle intermediate succinate.

Pathway‐level analyses presented below revealed consistent associations between metabolite ratios and the longevity gradient across central metabolic pathways (Figures 2, 3, 4, 5, 6 and Figures S2–S7). These patterns were supported by both univariate analyses and regression‐based approaches, highlighting a coherent set of metabolites associated with lifespan. To assess the influence of the exceptionally long‐lived *A. islandica* , all OLR analyses were repeated after excluding this species. Despite the resulting reduction in lifespan range and statistical power, the principal metabolic trends remained evident, although statistical support for some individual associations was reduced, particularly for several TCA‐cycle associations (Table S4).

### 3.2 Longevity‐Associated Remodeling of TCA Cycle Dynamics

Longevity was associated with marked shifts in TCA metabolite ratios, indicating changes in the relative organization of cycle intermediates in both gill and mantle tissues (Figures 2 and 6; Figure S4). The total metabolite content decreased along the longevity gradient only in gills (OLR *p* < 0.001, Figure 2A), while the trend was nearly significant in mantle tissue (OLR *p* = 0.057, Figure 2G). The sum of TCA intermediates differed significantly between species (ANOVA *p* = 0.003 in gills and *p* = 0.016 in mantle; Figure S3) but did not exhibit a monotonic pattern with lifespan (OLR *p* = 0.903 in gills and *p* = 0.223 in mantle; Figure 2B,H). The succinate/fumarate ratio increased significantly with longevity in both gills (OLR *p* = 0.002; Figure 2C) and mantle (OLR *p* < 0.001; Figure 2I), indicating a relative accumulation of succinate over fumarate. In mantle tissue, the malate/fumarate ratio also increased with longevity (OLR *p* = 0.004; Figure 2J), further supporting a redistribution of the intermediates within this region of the cycle. The pyruvate/malate ratio increased markedly with longevity in both tissues (OLR gills: *p* = 0.002; mantle: *p* = 0.006; Figure 2E,K), consistent with altered coupling between pyruvate metabolism and TCA cycle activity.

**FIGURE 2:**
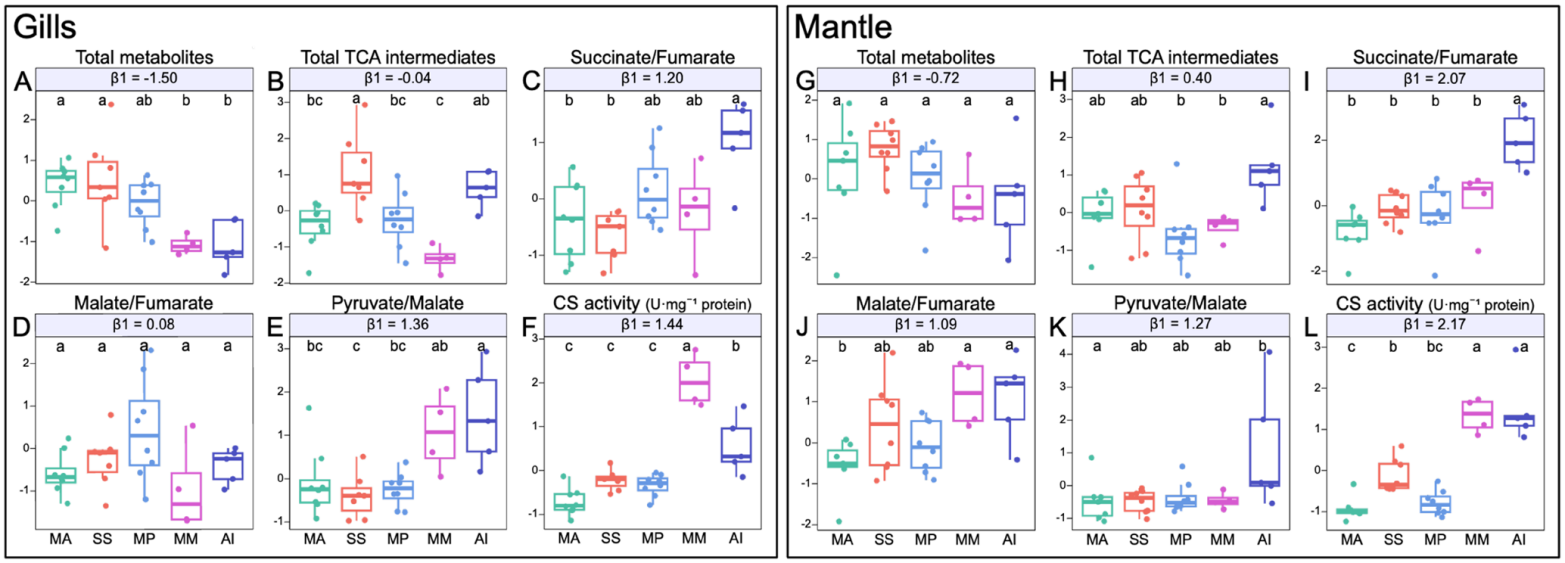
Total metabolite abundance, total tricarboxylic acid (TCA) cycle intermediates, metabolite ratios and citrate synthase (CS) activity standardized to protein in gills (A–F) and mantle (G–L) tissues across five bivalve species: *M. arenaria* (MA), *S. solidissima* (SS), *M. polynyma* (MP), *M. mercenaria* (MM), *A. islandica* (AI), ordered by increasing lifespan. Boxplots show the 25th (Q1) and 75th (Q3) percentiles (box limits) and median (center line). Letters indicate significant differences among species (ANOVA, *p* < 0.05, Tukey's post hoc test; or Kruskal–Wallis with Dunn's test when parametric assumptions were not met).

These ratio‐based changes were accompanied by coordinated shifts in individual metabolites. Fumarate and malate decreased with longevity (OLR *p* < 0.010, in gills and *p* < 0.005 in mantle; Figure 6 and Figure S4), whereas succinate showed the opposite pattern, reaching its highest levels in the longest‐lived species in the mantle tissue (OLR *p* = 0.018; Figure 6B and Figure S4), while gill tissue showed a similar tendency in univariate analyses that was not supported by OLR (*p* = 0.101, Figure 6A).

To assess whether these changes reflected differences in mitochondrial content, citrate synthase (CS) activity was measured as a proxy (Larsen et al. 2012). CS activity, normalized to protein content, was significantly higher in long‐lived species in both gills and mantle (OLR *p* < 0.001; Figure 2F,L). Among the TCA metabolites examined, only succinate exhibited a similar pattern (Figure 6). Together, these results suggest that longevity‐associated changes in TCA metabolites cannot be explained solely by increased mitochondrial content but instead reflect a reorganization of TCA cycle metabolite profiles.

### 3.3 Remodeling of Glycolytic Metabolites and Redistribution of Anaerobic Pyruvate Fates in Long‐Lived Species

The lactate/pyruvate ratio decreased markedly with increasing longevity in both tissues (OLR *p* < 0.001; Figure 3A,G), reflecting reduced lactate accumulation relative to pyruvate and suggesting altered pyruvate fate in long‐lived species. Lactate also showed a strong negative association with longevity in both tissues in the OLR analyses (*p* < 0.001, Figure 6 and Figure S5), further supporting a coordinated shift in glycolytic metabolites and downstream pyruvate metabolism in long‐lived species. Consistent with this shift, several nonessential AAs associated with anaerobic pyruvate metabolism also varied with lifespan. Strombine and octopine are two key anaerobic end‐products in bivalves (Müller et al. 2012). Glycine and arginine directly serve as substrates for strombine and octopine formation, respectively, whereas serine is metabolically connected through reversible conversion to glycine and broader links to glycolytic and redox metabolism. Substrate ratios involving these AAs showed strong negative relationships with lifespan, including serine/pyruvate and (glycine + arginine + alanine)/pyruvate in both tissues (OLR *p* < 0.018; Figure 3B,C,H,I). Ratios linking these substrates to their anaerobic end‐products also declined with lifespan, including (pyruvate + glycine)/strombine in both tissues (OLR *p* < 0.001; Figure 3D,J) and (pyruvate + arginine)/octopine in mantle tissue (OLR *p* < 0.001; Figure 3K), consistent with greater accumulation of strombine and octopine relative to their respective substrates in longer‐lived species.

**FIGURE 3:**
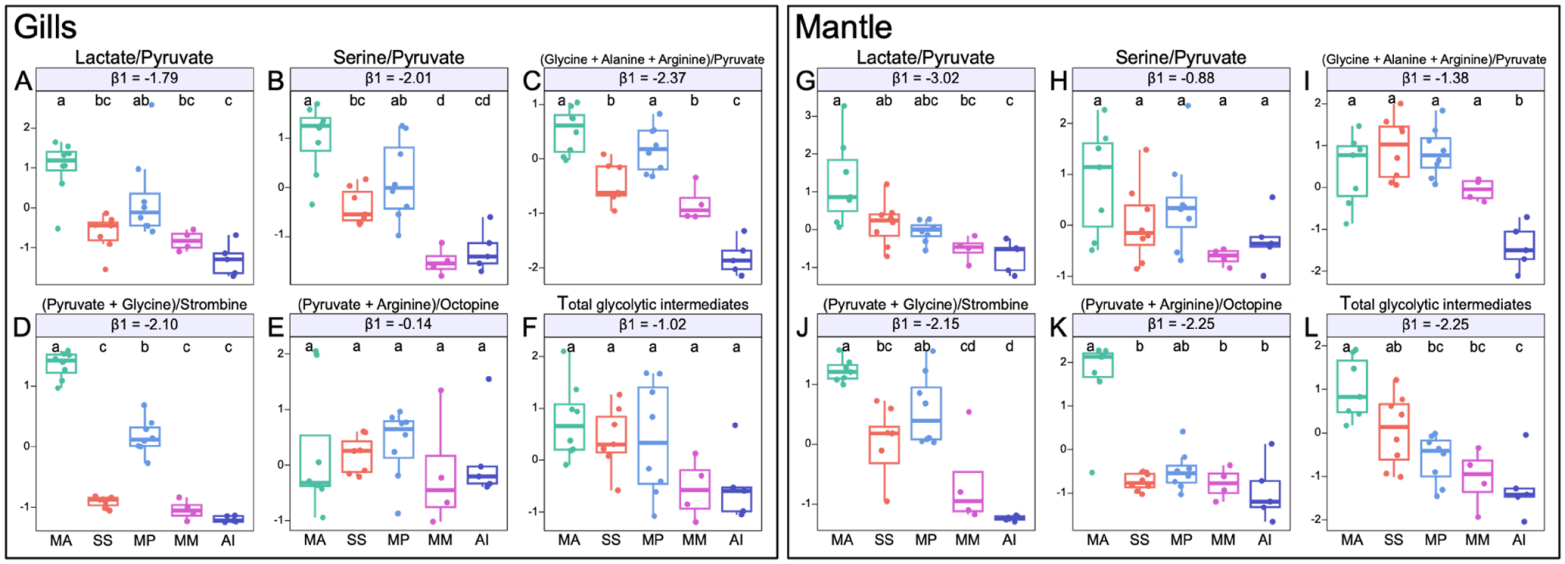
Metabolite ratios associated with glycolysis and anaerobic pyruvate metabolism in gill (A–F) and mantle (G–L) tissues across five bivalve species: *M. arenaria* (MA), *S. solidissima* (SS), *M. polynyma* (MP), *M. mercenaria* (MM), *A. islandica* (AI), ordered by increasing lifespan. Boxplots show the 25th (Q1) and 75th (Q3) percentiles (box limits) and median (center line). Data were log_10_‐transformed and autoscaled prior to analysis. Letters indicate significant differences among species (ANOVA, *p* < 0.05, Tukey's post hoc test; or Kruskal–Wallis with Dunn's test when parametric assumptions were not met).

In mantle tissue, upstream glycolytic intermediates (glucose, glucose‐6‐phosphate) showed negative associations with longevity (OLR *p* = 0.002 and *p* = 0.009, respectively; Figure 6B and Figure S5), whereas downstream intermediates were not associated with longevity (OLR: fructose‐1,6‐bisphosphate (F1, 6‐bP), *p* = 0.267; phosphoenolpyruvate (PEP), *p* = 0.843; Figure 6B and Figure S5). In contrast, gill tissues exhibited a different pattern, with no association of upstream glycolytic intermediates with longevity (OLR: glucose, *p* = 0.590; G6P, *p* = 0.504; Figure 6A and Figure S5), while F1, 6‐bP, an intermediate linked to the key glycolytic regulatory node (phosphofructokinase‐1, PFK‐1), showed a negative association with longevity (OLR *p* = 0.015; Figure 6A and Figure S5). Together, these data suggest tissue‐specific modifications in glycolytic regulation, involving glucose phosphorylation dynamics in mantle tissue and regulation at the PFK‐1 step in gill tissue. Summed concentrations of glycolytic metabolites did not vary significantly among species in gill tissue (*p* = 0.590, Figure 3F), whereas mantle tissue showed a longevity‐associated decline in total glycolytic metabolite concentrations (*p* < 0.001; Figure 3L).

### 3.4 Amino Acid Metabolism as a Central Signature of Longevity

In gill tissue, the significant decline in total metabolite content along the longevity gradient (OLR *p* < 0.001; Figure 2A) was largely driven by reductions in total free AA concentrations (OLR *p* < 0,001; Figure 4A). In the mantle tissue, total free AA levels also showed a significant negative association with longevity (OLR *p* = 0.010; Figure 4F), although the pattern was less clearly resolved, likely due to greater inter‐individual variability. Protein content normalized to tissue wet mass did not exhibit a consistent relationship with species longevity based on either post hoc comparison (Figure 4B,G), although OLR analyses were significant in both tissues (gills: *p* = 0.009; mantle: *p* = 0.009). This discrepancy likely reflects interspecific differences that were not consistently ordered along the longevity gradient. Overall, these results indicate that the reduced free AA levels observed in long‐lived species are not simply attributable to differences in total protein abundance.

**FIGURE 4:**
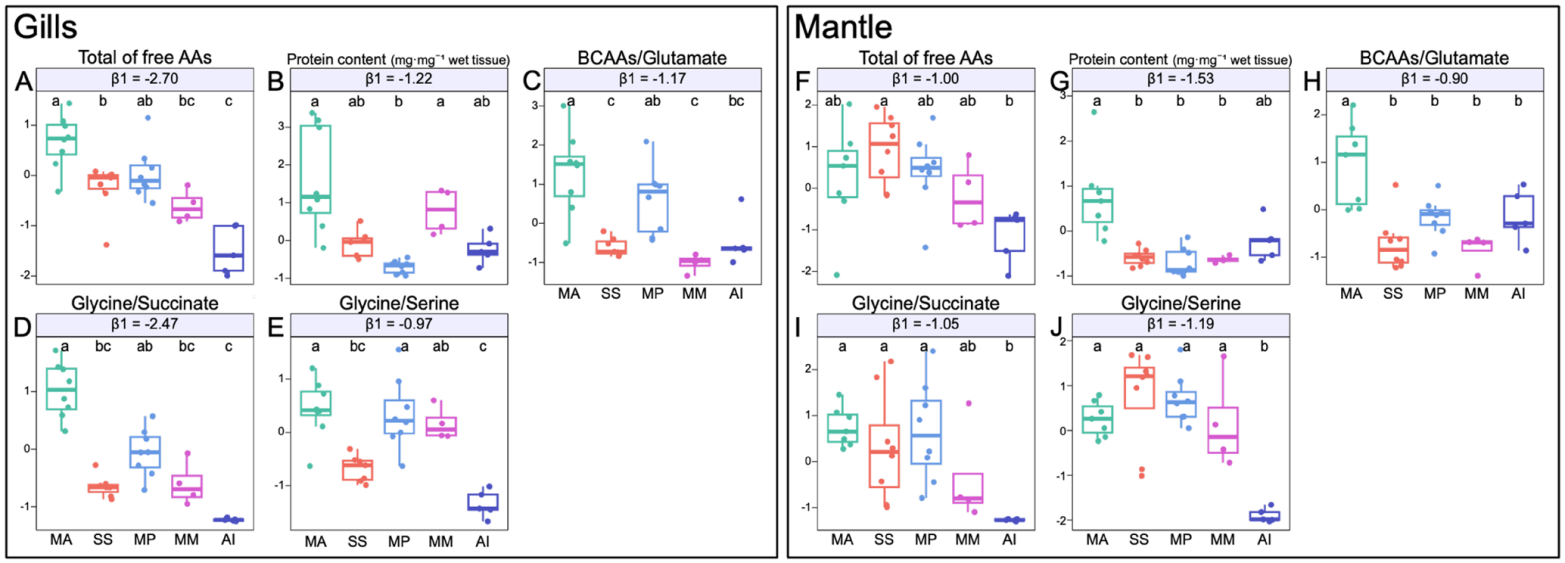
Total ratio amino acids and protein content standardized to wet tissue mass in gill (A–E) and mantle (F–J) tissues across five bivalve species: *M. arenaria* (MA), *S. solidissima* (SS), *M. polynyma* (MP), *M. mercenaria* (MM), *A. islandica* (AI), ordered by increasing lifespan. Boxplots show the 25th (Q1) and 75th (Q3) percentiles (box limits) and median (center line). Data were log_10_‐transformed and autoscaled prior to analysis. Letters indicate significant differences among species (ANOVA, *p* < 0.05, Tukey's post hoc test; or Kruskal–Wallis with Dunn's test when parametric assumptions were not met).

Beyond these global reductions in the free AA pool, several AAs linked to anaplerotic fueling of central carbon metabolism displayed tissue‐specific associations with longevity. In gills, the ratio of total BCAAs (leucine + valine + isoleucine) to glutamate varied among species and decreased along the longevity gradient (OLR *p* = 0.004; Figure 4C). Consistent with their role as ketogenic and glucogenic substrates, BCAAs, as well as threonine, displayed strong negative *β*_1_ values in gill tissue and were among the metabolites most strongly associated with species longevity (OLR *p* ≤ 0.001; Figure 6A and Figure S6). However, when normalized to total AAs, BCAA differences disappeared (Figure S6). In mantle tissue, neither the total BCAAs‐to‐glutamate ratio (Figure 4H) nor individual BCAAs (Figure 6B and Figure S6) showed comparable associations with longevity, highlighting tissue‐specific remodeling of AA metabolism in gills.

Beyond BCAAs, several additional AAs involved in anaplerotic and biosynthetic metabolism showed strong associations with longevity. Glycine showed a marked negative association with lifespan in both tissues (OLR *p* < 0.001, Figure 6 and Figure S5). Serine and alanine exhibited similar patterns in both tissues, with stronger negative *β*_1_ values in gills than in mantle tissue (OLR *p* < 0.005; Figure 6 and Figure S5). Arginine also decreased with longevity in mantle tissue (OLR *p* < 0.001, Figure 6 and Figure S5). The glycine/succinate ratio declined significantly with increasing longevity in both tissues (OLR *p* < 0.001 in gills and *p* = 0.005 in mantle; Figure 4D,I), reflecting the combined decrease in glycine and relative increase in succinate concentrations along the lifespan gradient, suggesting a shift in the balance between free AA pools and TCA intermediates with increasing longevity. Similarly, the glycine/serine ratio declined markedly with lifespan (OLR *p* = 0.012 in gills and *p* = 0.003 in mantle; Figure 4E,J). Because glycine and serine are interconvertible through serine hydroxymethyltransferase, this ratio is consistent with remodeling of one‐carbon metabolism and methyl‐group transfer pathways in long‐lived species. Consistent with these shifts in one‐carbon and AA metabolism, methionine also showed a negative association with longevity, particularly in gill tissue (OLR *p* < 0.001 in gills and *p* = 0.031 in mantle; Figure 6 and Figure S6). Importantly, these associations observed for individual AAs remained significant after normalization to total AA content (Figures S5 and S6).

### 3.5 Cellular Energy Charge and Redox State Remain Stable Across the Longevity Gradient

Indicators of cellular energy status and redox cofactors did not display a clear pattern along the longevity gradient in either tissue. Key adenylate ratios, including adenylate energy charge (AEC: (ATP + 0.5 × ADP)/(ATP + ADP + AMP)), showed no significant association with lifespan, although weak positive associations were detected for AMP/ATP and ADP/ATP ratios in gill tissues (OLR *p* < 0.05; Figure 5A–C,E–G). The NADP^+^/NADPH ratio, an indicator of cellular reductive capacity, remained stable along the longevity gradient in mantle (OLR *p* = 0.642, Figure 5H), whereas a slight decrease was observed in gill tissues (OLR *p* < 0.005; Figure 5D).

**FIGURE 5:**
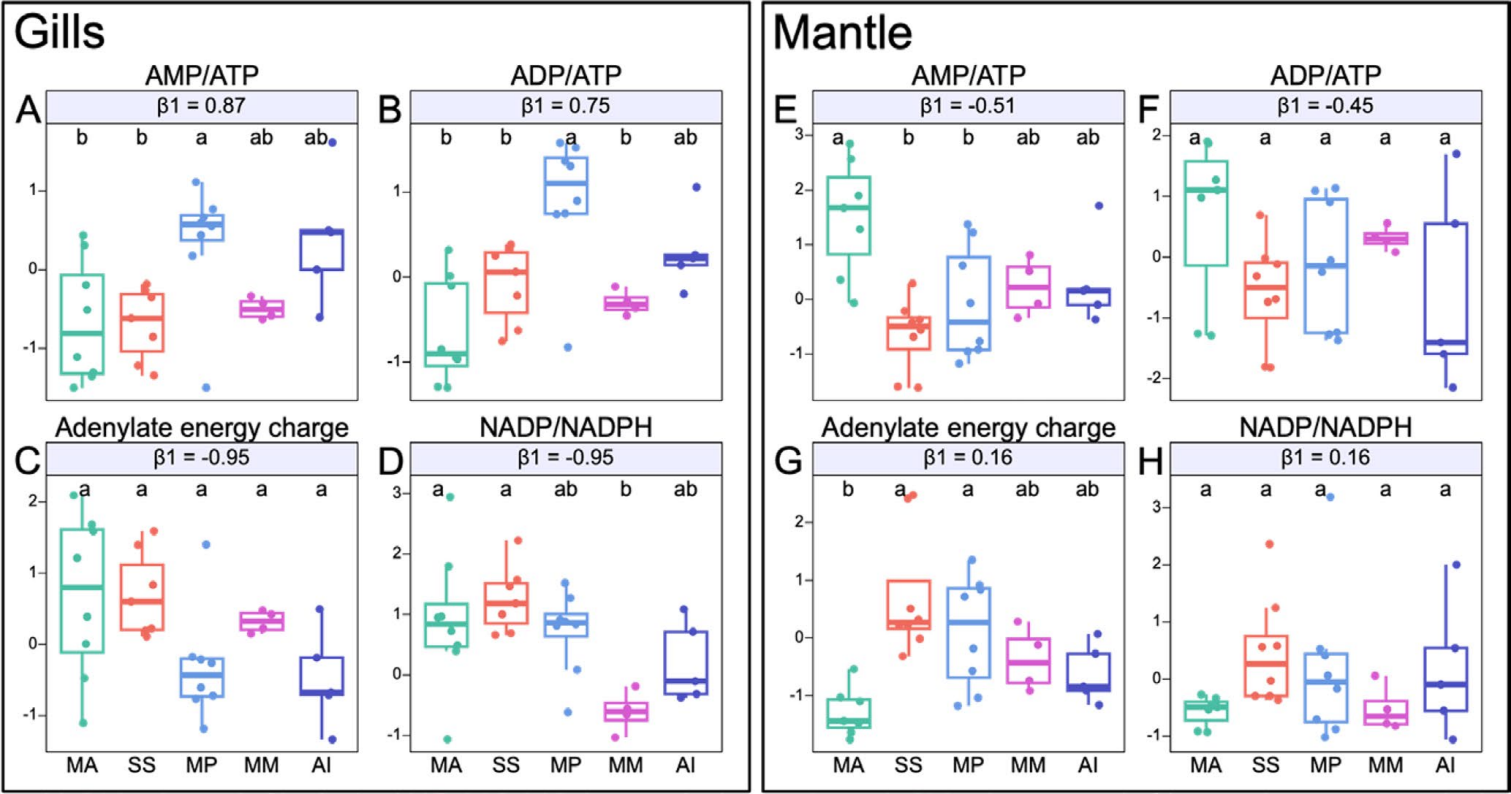
Energy cofactors ratios in gill (A–D) and mantle (E–H) tissues across five bivalve species: *M. arenaria* (MA), *S. solidissima* (SS), *M. polynyma* (MP), *M. mercenaria* (MM), *A. islandica* (AI), ordered by increasing lifespan. Boxplots show the 25th (Q1) and 75th (Q3) percentiles (box limits) and median (center line). Data were log_10_‐transformed and autoscaled prior to analysis. Letters indicate significant differences among species (ANOVA, *p* < 0.05, Tukey's post hoc test; or Kruskal–Wallis with Dunn's test when parametric assumptions were not met).

**FIGURE 6:**
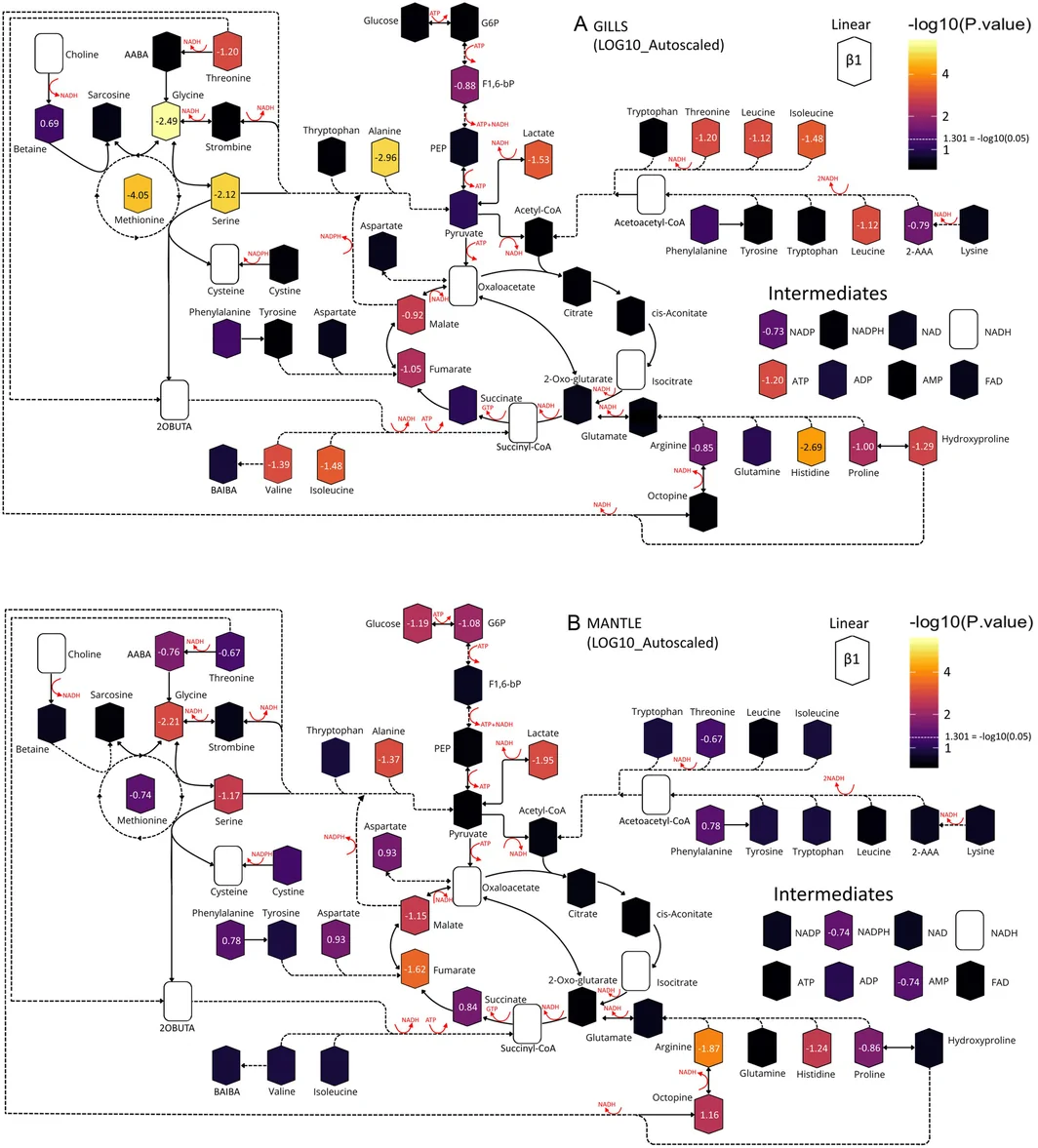
Metabolic map illustrating glycolysis, the tricarboxylic acid (TCA) cycle, and the entry points of amino acids and related intermediates into central carbon metabolism in gill (A) and mantle (B) tissues, organized by pathway with glycolysis and the TCA cycle centrally positioned, the methionine cycle on the left, and amino acid pathways arranged according to their entry points. Each metabolite is represented according to the results of the ordinal logistic regression (proportional odds model). The estimated linear coefficient $\beta_{1}$ is shown within each shape. The fill color represents statistical significance (−log_10_ *p*‐value), with –log_10_(0.05) = 1.301 as the significance threshold, and lighter colours indicating the highest statistical significance. Uncolored boxes correspond to metabolites not measured but included for completeness of the metabolic map. Continuous lines indicate direct reactions between metabolites, whereas dashed lines represent multi‐step reaction sequence. Cofactors involved in specific pathways are shown in red.

## 4 Discussion

Longevity in animals arises from a finely tuned balance among mitochondrial efficiency, cellular maintenance, and resistance to environmental and physiological stressors (López‐Otín et al. 2023). We identify a metabolomic signature associated with exceptional longevity across the bivalve species examined, highlighting a central role for mitochondrial metabolism. Pronounced species‐specific metabolomic profiles aligned closely with maximum reported lifespan across both gill and mantle tissues. However, lifespan‐associated variation was distributed across distinct metabolic dimensions in each tissue, and each tissue appeared to capture different aspects of the organism's metabolic strategy, reflecting its physiological role and environmental exposure. Gill tissue more clearly resolved lifespan‐associated patterns than mantle tissue, potentially owing to its higher metabolic activity, direct interaction with the external environment, and possible role in managing oxidative challenges associated with repeated hypoxia‐reoxygenation events, whereas the mantle reflects greater cellular heterogeneity and functional complexity (Gosling 2015). The consistency of these observations across multiple analytical approaches supports the hypothesis of a coordinated metabolic program associated with extended longevity in bivalves. Similar longevity‐associated metabolic changes have been reported in vertebrates and other animal models (reviewed in Parkhitko et al. 2020), suggesting that some of these features may represent evolutionarily conserved components of metabolic adaptation to extended lifespan.

Longevity‐associated changes were not uniformly distributed across the TCA cycle, as several upstream intermediates remained relatively stable. This pattern suggests that longevity‐related metabolic reorganization is localized to specific regulatory nodes of the TCA cycle rather than reflecting a generalized reduction in TCA activity. The increased succinate‐to‐fumarate ratio, together with increased malate‐to‐fumarate ratios in mantle, suggests a redistribution of metabolites around the succinate dehydrogenase–fumarase node. This pattern is consistent with previous data showing reduced succinate dehydrogenase (SDH) activity and lower CII control strength in long‐lived bivalves (Munro et al. 2013; Rodríguez et al. 2021), suggesting that electron entry through the succinate‐linked pathway is selectively reorganized with longevity. Succinate emerged as a central metabolite in this metabolic remodeling. Beyond its role as a TCA intermediate, succinate acts as an important signaling molecule that inhibits α‐ketoglutarate‐dependent dioxygenases and stabilizes hypoxia‐inducible factor‐1α (HIF‐1α), thereby promoting a pseudo‐hypoxic transcriptional response linked to metabolic flexibility and stress tolerance (Murphy and Chouchani 2022; Kuo et al. 2022). Together, these findings suggest that succinate occupies a key position at the interface of energy metabolism, redox signaling, and cellular stress responses, potentially contributing to metabolic adaptations associated with exceptional longevity in bivalves.

Because fumarate, malate, and succinate are interconnected through multiple reversible and anaplerotic reactions, these changes likely reflect coordinated regulation of this region of the TCA cycle rather than altered activity of a single enzyme. Fumarate and malate concentrations declined with increasing lifespan in both tissues. In mantle tissue, the proportionally greater reduction in fumarate resulted in an increased malate‐to‐fumarate ratio, whereas parallel reductions in both metabolites maintained a relatively stable ratio in gills. Such tissue‐specific patterns may reflect functional specialization, particularly because gills directly interface with environmental fluctuations and oxidative challenges (Gosling 1992) and undergo constant cellular turnover. These changes around the succinate–fumarate node may also have implications for mitochondrial redox homeostasis, particularly during hypoxia–reoxygenation. Under oxygen‐limited conditions, fumarate can serve as an alternative terminal electron acceptor and be reduced to succinate through reverse succinate dehydrogenase (SDH) activity (fumarate reductase activity), thereby supporting mitochondrial function when oxygen availability is limited (Spinelli et al. 2021). Conversely, during reoxygenation, reduced succinate oxidation may limit over‐reduction of the coenzyme Q pool and attenuate reverse electron transport (RET) at CI, a major source of mitochondrial ROS production (Chouchani et al. 2014; Quinlan et al. 2012; Scialò et al. 2016). Interestingly, reduced RET upon reoxygenation has recently been linked to lower CII‐supported respiration in the exceptionally long‐lived and hypoxia‐tolerant naked mole‐rat (Munro et al. 2026), paralleling the lower SDH activity previously reported in the long‐lived and hypoxia‐tolerant *Arctica islandica* (Munro et al. 2013). Although our metabolomic data do not establish the direction of SDH activity, the increase in succinate‐to‐fumarate ratios with longevity raises the possibility that regulation of this metabolic node may represent a conserved metabolic strategy linking mitochondrial electron flow, adaptation to oxygen availability, and longevity.

In parallel, the increase in the pyruvate‐to‐malate ratio suggests altered coupling between pyruvate utilization and TCA cycle metabolism, potentially reflecting changes in mitochondrial redox balance and NADH‐dependent metabolism. Consistent with this interpretation, reduced CI activity or a lower relative contribution of the NADH pathway has previously been associated with longevity across multiple taxa, including bivalves (Munro et al. 2013; Rodríguez et al. 2021; Dillin et al. 2002). Together with the succinate‐associated changes described above, these ratio‐based signatures are consistent with differences in electron entry into the mitochondrial respiratory system through both NADH‐ and succinate‐linked pathways in long‐lived species. Importantly, citrate synthase activity increased with longevity, indicating that these metabolic changes are unlikely to arise from reduced mitochondrial abundance. Instead, the dissociation between mitochondrial content and metabolite organization, with only succinate showing a positive relationship with CS activity, suggests functional metabolic remodeling rather than simple scaling of TCA intermediates with mitochondrial content. Taken together, these findings may support a model in which exceptional longevity is associated with coordinated reorganization of TCA cycle dynamics and its integration with the ETS. Rather than reflecting a generalized suppression of metabolism, this configuration may represent a shift toward a more controlled and balanced electron supply to the ETS, potentially contributing to reduced oxidative burden and the long‐term preservation of mitochondrial function.

Bivalves possess a broader anaerobic metabolic repertoire than vertebrates, producing the opine metabolites strombine, octopine and alanopine, in addition to lactate (de Zwaan and Wijsman 1976). Across both tissues, long‐lived species displayed convergent metabolic signatures characterized by: (1) reduced glycolytic intermediates, with effects differing between gill and mantle tissue; (2) lower lactate‐to‐pyruvate ratios accompanied by decreased alanine and glycine concentrations, with lactate showing strong negative associations with longevity; (3) reduced arginine levels in mantle; (4) unchanged opine levels; and (5) consistent reductions in pyruvate‐related ratios including glycine/pyruvate, alanine/pyruvate, and derived opine ratios. Rather than indicating a loss of anaerobic capacity, these patterns may reflect tighter regulation of anaerobic metabolism during routine physiological conditions. Long‐lived species therefore appear to maintain a metabolic state consistent with reduced engagement of anaerobic pathways during basal metabolism. Such an organization could reduce unnecessary metabolite accumulation while preserving metabolic flexibility for rapid recruitment under oxygen limitation.

Among amino acids, glycine and serine emerged as strong and consistent predictors of longevity across tissues and statistical approaches. Glycine, one of the most abundant metabolites in both tissues (Ellis et al. 1985), together with serine, participates in one‐carbon metabolism through the interconversion by serine hydroxymethyltransferase. This pathway feeds into the tetrahydrofolate cycle and supports nucleotide biosynthesis, as well as the regeneration of methionine from homocysteine via the S‐adenosylmethionine/S‐adenosylhomocysteine (SAM/SAH) methylation cycle, thereby influencing genomic and epigenetic stability (Lopes 2020). Glycine also serves as a direct precursor of glutathione (GSH), a major intracellular antioxidant, whereas serine contributes indirectly through its conversion into glycine (Lu 2013). Studies in mammals have reported enhanced antioxidant capacity in long‐lived individuals, accompanied by increased availability of glycine and other GSH‐related amino acids (Yu et al. 2025). Consistent with these observations, *A. islandica* relies predominantly on GSH‐dependent mitochondrial H_2_O_2_ detoxification and exhibits substantially greater antioxidant capacity than short‐lived species (Munro et al. 2023). Consequently, the reduced glycine and serine levels observed in long‐lived bivalves may reflect an increased demand rather than reduced importance, potentially supporting GSH synthesis and one‐carbon metabolic activity. Such redistribution may contribute to sustained antioxidant defense and maintenance of redox balance, particularly under fluctuating oxygen conditions. The reduced glycine‐to‐serine ratio in long‐lived species further suggests a shift in one‐carbon metabolism rather than a simple limitation in substrate availability. This pattern may reflect a more tightly regulated epigenomic state, a process increasingly linked to aging and associated with lifespan variation (Annibal et al. 2021; Liu et al. 2019).

Methionine also displays a strong negative relationship with longevity and contributed substantially to species discrimination. This pattern is consistent with comparative studies in mammals linking methionine metabolism to species longevity (Ruiz et al. 2005; Mota‐Martorell et al. 2021). Across multiple animal models, methionine restriction has been associated with lifespan extension through suppression of mTORC1 signaling and downstream IGF‐1 pathways (Tatar et al. 2025; Gu et al. 2017). In addition, short‐lived mammals show greater methionine content in mtDNA‐encoded proteins, a pattern proposed to reflect adaptation to elevated oxidative burden, whereas long‐lived species show reduced methionine incorporation, potentially consistent with lower ROS production or enhanced ROS buffering capacity (Aledo et al. 2011). Together, these parallels suggest that the association between methionine metabolism, nutrient sensing, and longevity may represent an evolutionarily conserved feature across animal taxa (reviewed in Kitada et al. 2021).

Betaine, one of the dominant osmolytes in marine bivalves (de Vooys and Geenevasen 2002), was among the most abundant metabolites in both tissues. Beyond its role in osmotic regulation, betaine serves as a direct methyl donor in methionine regeneration, bypassing folate‐dependent pathways and functionally linking methyl metabolism with cellular energetics (Lever and Slow 2010). Consistent with this multifunctional role, the betaine‐to–total AA ratio increased with longevity in both tissues. Increased reliance on betaine may therefore provide a more direct route for sustaining methylation capacity, potentially reducing dependence on glycine and serine turnover while supporting energetic stability and cellular resilience under chronic environmental and metabolic stress. Branched‐chain amino acids (BCAAs) also declined progressively along the longevity gradient in gill tissue. Because BCAAs are established activators of mTOR signaling, their reduction is broadly consistent with diminished activity of pathways associated with accelerated aging (Mansfeld et al. 2015; Cavigliasso et al. 2023). However, normalization to total AA content eliminated these differences, suggesting that reductions in BCAAs largely reflect a broader contraction of the free AA pool. Nevertheless, the decreasing BCAAs‐to‐glutamate ratio suggests relative preservation of glutamate as a central metabolic hub (Brosnan and Brosnan 2013), indicating that AA metabolism undergoes selective reorganization rather than uniform depletion. More broadly, amino acid metabolism is increasingly recognized as an important regulator of aging and longevity (Torres et al. 2023). Consistent with findings across taxa (Lewis et al. 2018; Yu et al. 2025), the total free AA pool declined significantly along the longevity gradient in gills and showed a similar, though more variable, tendency in mantle tissue. Together, these findings suggest a refined AA economy in long‐lived species, potentially reducing investment in growth‐associated processes while preserving pathways involved in cellular maintenance and stress resistance.

Because *M. arenaria* experiences recurrent hypoxia–reoxygenation cycles and osmotic stress inherent to intertidal life, we expected that these distinctive ecological constraints might influence some metabolic traits independently of longevity. Nevertheless, *M. arenaria* followed a general longevity‐associated pattern for most metabolites, with only a limited number of exceptions. An exploratory analysis excluding this species strengthened several longevity‐associated trends, including those for aspartate and the G6P/glucose ratio, α‐ketoglutarate levels and the α‐ketoglutarate/glutamate ratio in mantle tissue, as well as phenylalanine, tyrosine, and betaine content, including after normalization to total amino acids. These observations should be considered hypothesis‐generating and highlight metabolic traits for which ecological adaptation should be examined alongside longevity in future comparative studies. At the opposite end of the longevity gradient, many of the principal metabolic patterns also persisted after exclusion of the exceptionally long‐lived *A. islandica* , including associations involving amino acid and anaerobic pyruvate metabolism, as well as TCA‐cycle metabolites, particularly fumarate and malate. These results indicate that the overall metabolic signature was not solely driven by this extreme endpoint.

In summary, our findings reveal tissue‐specific metabolomic associations with longevity across bivalve species. We observe a lifespan‐dependent remodeling of mitochondrial metabolism, particularly around the succinate‐fumarate node, coupled with broader changes in amino acids and pyruvate‐related metabolism. Rather than indicating a generalized metabolic slowdown in long‐lived species, these changes may suggest a redistribution of metabolic resources toward maintaining redox balance, preserving mitochondrial function, and enhancing resilience to environmental stressors.

An important avenue for future work will be to determine whether lifespan variation among bivalves covaries with their capacity to tolerate and recover from hypoxia. Marine bivalves experience recurrent hypoxic episodes, and succinate‐centred metabolic remodeling represents a conserved response to hypoxia, heat stress, salinity fluctuations, and other environmental challenges (de Zwaan and Wijsman 1976; Hu et al. 2022; Orita et al. 2025; Sokolova 2021). Long‐lived bivalves such as *A. islandica* and *M. mercenaria* exhibit exceptional hypoxia tolerance (Steffen et al. 2021; Strahl et al. 2011), while emerging evidence from other long‐lived systems suggests a link between succinate‐supported mitochondrial metabolism and whole‐organism hypoxia tolerance (Munro et al. 2026). Together, these observations raise the possibility that the metabolic architecture associated with longevity supports both long‐term cellular maintenance and adaptation to environmental stress, contributing to the remarkable ability of long‐lived bivalves to maintain homeostasis across exceptionally long lifespans.

## Author Contributions

Pauline Bertrand conceived the project, established and maintained the aquaculture system, acquired and maintained the study animals, performed tissue sampling, metabolomic and enzymatic analyses, conducted statistical analyses, interpreted the data, prepared the figures, and wrote the first draft of the manuscript. Clément J.L. Chevret developed the ordinal logistic regression approach, contributed to data interpretation, and participated in manuscript preparation and revision. Hélène Lemieux contributed to data interpretation and made substantial contributions to the drafting, critical revision, and finalization of the manuscript. Pierre U. Blier conceived the project, secured funding, contributed to data interpretation and participated in manuscript redaction and revision. Enrique Rodriguez participated in manuscript revision.

## Funding

This study was supported by the following grants to P.U.B.: a discovery grant from the Natural Sciences and Engineering Research Council of Canada (RGPIN 2019‐05992).

## Conflicts of Interest

The authors declare no conflicts of interest.

## Supporting information


**Table S1:** Summary of PERMANOVA results testing the effects of species, tissue, species × tissue interaction, and body size (covariate) on metabolomic profiles in gill and mantle tissues, including pairwise post hoc species comparisons within each tissue.
**Table S2:** Metabolites with Pearson correlation coefficient ≥ 0.70 from PCA on gill and mantle metabolomic data. Bold component labels indicate significant correlations relative to MRL, and gray shading highlights metabolites with VIP score > 1 in comp. 1 of PLS‐DA, underscoring their importance.
**Table S3:** Variable importance in projection (VIP) scores calculated from the first three components of PLS‐DA, performed separately for gill and mantle tissues, highlighting the metabolites contributing most to species discrimination (VIP score > 1). Gray shading highlights metabolites Pearson correlation coefficient ≥ 0.70 from PCA.
**Table S4:** Ordinal logistic regression (OLR) results for all metabolites in gill (A) and mantle (B) tissues. For each metabolite, the regression coefficient (*β*_1_), *p*‐value, Benjamini‐Hochberg adjusted *p*‐value (FDR correction), Brant test and permutation test results are reported. Corresponding analyses performed after exclusion of the exceptionally long‐lived *A. islandica* are also provided.
**Figure S1:** Partial least squares–discriminant analysis (PLS‐DA) of metabolite profiles across five bivalve species: *M. arenaria* (MA), *S. solidissima* (SS), *M. polynyma* (MP), *M. mercenaria* (MM), *A. islandica* (AI). Score plots of the first two components with 95% confidence ellipses are shown for gills (A) and mantle (B). The arrow indicates the direction of the longevity gradient. VIP scores on the right‐hand panels indicate the metabolites most important for species discrimination (VIP > 1), and heatmaps display the relative concentration of each metabolite across species with increasing MRL.
**Figure S2:** Barplots showing the significance of interspecific differences for each metabolite in gills (A) and mantle (B). Bars represent –log10 of adjusted *p*‐values (FDR correction), with the dashed red line indicating the significance threshold (*p* < 0.05). Bar colors correspond to log10‐transformed mean raw concentrations of metabolites (see color scale on the right). Statistical tests were performed using ANOVA with Tukey's post hoc test, or Kruskal–Wallis with Dunn's test when parametric assumptions were not met; metabolites analyzed with the Kruskal–Wallis test are marked with a triangle next to their name on the x‐axis.
**Figure S3:** Barplots showing the significance of interspecific differences for each ratio in gills (A) and mantle (B). Bars represent –log10 of adjusted *p*‐values (FDR correction), with the dashed red line indicating the significance threshold (*p* < 0.05). Bar colors correspond to the statistical test applied: ANOVA with Tukey's post hoc test or Kruskal–Wallis with Dunn's test (see color scale on the right).
**Figure S4:** Boxplots showing TCA cycle‐related metabolites and ratios in gill (A) and mantle (B) tissues across five bivalve species: *M. arenaria* (MA), *S. solidissima* (SS), *M. polynyma* (MP), *M. mercenaria* (MM), *A. islandica* (AI), ordered by increasing lifespan. Boxplots show the 25th (Q1) and 75th (Q3) percentiles (box limits) and median (center line). Data were log_10_‐transformed and autoscaled prior to analysis. Letters indicate significant differences among species (ANOVA, *p* < 0.05, Tukey's post hoc test; or Kruskal–Wallis with Dunn's test when parametric assumptions were not met).
**Figure S5:** Boxplot showing glycolytic and anaerobic pyruvate metabolism in gill (A) and mantle (B) tissues across five bivalve species: *M. arenaria* (MA), *S. solidissima* (SS), *M. polynyma* (MP), *M. mercenaria* (MM), *A. islandica* (AI), ordered by increasing lifespan. Boxplots show the 25th (Q1) and 75th (Q3) percentiles (box limits) and median (center line). Data were log_10_‐transformed and autoscaled prior to analysis. Letters indicate significant differences among species (ANOVA, *p* < 0.05, Tukey's post hoc test; or Kruskal–Wallis with Dunn's test when parametric assumptions were not met).
**Figure S6:** Boxplot showing amino acids metabolites and associated ratios in gill (A) and mantle (B) tissues across five bivalve species: *M. arenaria* (MA), *S. solidissima* (SS), *M. polynyma* (MP), *M. mercenaria* (MM), *A. islandica* (AI), ordered by increasing lifespan. Boxplots show the 25th (Q1) and 75th (Q3) percentiles (box limits) and median (center line). Data were log_10_‐transformed and autoscaled prior to analysis. Letters indicate significant differences among species (ANOVA, *p* < 0.05, Tukey's post hoc test; or Kruskal–Wallis with Dunn's test when parametric assumptions were not met).
**Figure S7:** Boxplot showing energetic cofactors and total energetic cofactors abundance in gill (A) and mantle (B) tissues across five bivalve species: *M. arenaria* (MA), *S. solidissima* (SS), *M. polynyma* (MP), *M. mercenaria* (MM), *A. islandica* (AI), ordered by increasing lifespan. Boxplots show the 25th (Q1) and 75th (Q3) percentiles (box limits) and median (center line). Data were log_10_‐transformed and autoscaled prior to analysis. Letters indicate significant differences among species (ANOVA, *p* < 0.05, Tukey's post hoc test; or Kruskal–Wallis with Dunn's test when parametric assumptions were not met).

**Data S2:** acel70772‐sup‐0002‐DataS2.docx.

## Data Availability

The raw data supporting the findings of this study will be made publicly available in Borealis, the Canadian Dataverse Repository, upon publication.
