# Supplementary material for: Metabolomic Signatures of Longevity in Bivalves Reveal Reorganization of TCA Cycle and Amino Acid Metabolism
Source: Aging Cell. 2026 Oct 25;25(11):e70772. doi: 10.1111/acel.70772 (PMC13696439; doi:10.1111/acel.70772)
Supplement: Supplementary file 1 [file ACEL-25-e70772-s001.docx]

**Supplementary Material**

**Table S1** Summary of PERMANOVA results testing the effects of species, tissue, species × tissue interaction, and body size (covariate) on metabolomic profiles in gill and mantle tissues, including pairwise post hoc species comparisons within each tissue.

Analysis based on a Euclidean distance matrix, with 999 permutations. DF: degrees of freedom; R²: proportion of variance explained. Pairwise *P*-values were adjusted for multiple comparisons using the Benjamini–Hochberg FDR correction, with significant adjusted values (*p* < 0.05) shown in bold. Significance codes: '***' *p < 0.001,* '**' *p < 0.01,* '*' *p* < 0.05. *M. arenaria* (MA), *S. solidissima* (SS), *M. polynyma* (MP), *M. mercenaria* (MM), *A. islandica* (AI).

**Table S2** Metabolites with Pearson correlation coefficient ≥ 0.70 from PCA on gills and mantle metabolomic data. Bold component labels indicate significant correlations relative to MRL, and grey shading highlights metabolites with VIP score > 1 in comp. 1 of PLS-DA, underscoring their importance.

 **Figure S1** Partial least squares–discriminant analysis (PLS-DA) of metabolite profiles across five bivalve species: *M. arenaria* (MA), *S. solidissima* (SS), *M. polynyma* (MP), *M. mercenaria* (MM), *A. islandica* (AI). Score plots of the first two components with 95% confidence ellipses are shown for gills (A) and mantle (B). The arrow indicates the direction of the longevity gradient. VIP scores on the right-hand panels indicate the metabolites most important for species discrimination (VIP > 1), and heatmaps display the relative concentration of each metabolite across species with increasing MRL.

**Table S3** Variable importance in projection (VIP) scores calculated from the first three components of PLS-DA, performed separately for gill and mantle tissues, highlighting the metabolites contributing most to species discrimination (VIP score > 1). Grey shading highlights metabolites Pearson correlation coefficient ≥ 0.70 from PCA.

**Figure S2** Barplots showing the significance of interspecific differences for each metabolite in gills (A) and mantle (B). Bars represent –log10 of adjusted p-values (FDR correction), with the dashed red line indicating the significance threshold (p < 0.05). Bar colors correspond to log10-transformed mean raw concentrations of metabolites (see color scale on the right). Statistical tests were performed using ANOVA with Tukey’s post-hoc test, or Kruskal–Wallis with Dunn’s test when parametric assumptions were not met; metabolites analyzed with the Kruskal–Wallis test are marked with a triangle next to their name on the x-axis.

**Figure S3** Barplots showing the significance of interspecific differences for each ratio in gills (A) and mantle (B). Bars represent –log10 of adjusted p-values (FDR correction), with the dashed red line indicating the significance threshold (p < 0.05). Bar colors correspond to the statistical test applied: ANOVA with Tukey’s post-hoc test (blue) or Kruskal–Wallis with Dunn’s test (grey).

**Figure S4 Boxplots showing TCA cycle-related metabolites and ratios** in gill (A) and mantle (B) tissues across five bivalve species: *M. arenaria* (MA), *S. solidissima* (SS), *M. polynyma* (MP), *M. mercenaria* (MM), *A. islandica* (AI), ordered by increasing lifespan. Boxplots show the 25th (Q1) and 75th (Q3) percentiles (box limits) and median (center line). Data were log₁₀-transformed and autoscaled prior to analysis. Letters indicate significant differences among species (ANOVA, p < 0.05, Tukey’s post-hoc test; or Kruskal–Wallis with Dunn’s test when parametric assumptions were not met).

**Figure S5 Boxplot showing glycolytic and anaerobic pyruvate metabolism** in gill (A) and mantle (B) tissues across five bivalve species: *M. arenaria* (MA), *S. solidissima* (SS), *M. polynyma* (MP), *M. mercenaria* (MM), *A. islandica* (AI), ordered by increasing lifespan. Boxplots show the 25th (Q1) and 75th (Q3) percentiles (box limits) and median (center line). Data were log₁₀-transformed and autoscaled prior to analysis. Letters indicate significant differences among species (ANOVA, p < 0.05, Tukey’s post-hoc test; or Kruskal–Wallis with Dunn’s test when parametric assumptions were not met).

**Figure S6 Boxplot showing amino acids metabolites and associated** ratios in gill (A) and mantle (B) tissues across five bivalve species: *M. arenaria* (MA), *S. solidissima* (SS), *M. polynyma* (MP), *M. mercenaria* (MM), *A. islandica* (AI), ordered by increasing lifespan. Boxplots show the 25th (Q1) and 75th (Q3) percentiles (box limits) and median (center line). Data were log₁₀-transformed and autoscaled prior to analysis. Letters indicate significant differences among species (ANOVA, p < 0.05, Tukey’s post-hoc test; or Kruskal–Wallis with Dunn’s test when parametric assumptions were not met).

**Figure S7 Boxplot showing energetic cofactors and total energetic cofactors abundance** in gill (A) and mantle (B) tissues across five bivalve species: *M. arenaria* (MA), *S. solidissima* (SS), *M. polynyma* (MP), *M. mercenaria* (MM), *A. islandica* (AI), ordered by increasing lifespan. Boxplots show the 25th (Q1) and 75th (Q3) percentiles (box limits) and median (center line). Data were log₁₀-transformed and autoscaled prior to analysis. Letters indicate significant differences among species (ANOVA, p < 0.05, Tukey’s post-hoc test; or Kruskal–Wallis with Dunn’s test when parametric assumptions were not met).

**Table S4** Ordinal logistic regression (OLR) results for all metabolites in gill (A) and mantle (B) tissues. For each metabolite, the regression coefficient (β₁), p-value, Benjamini-Hochberg adjusted p-value (FDR correction), Brant test and permutation test results are reported. Corresponding analyses performed after exclusion of the exceptionally long-lived A. islandica are also provided.
