# Supplementary material for: Metabolomic Signatures of Longevity in Bivalves Reveal Reorganization of TCA Cycle and Amino Acid Metabolism
Source: Aging Cell. 2026 Oct 25;25(11):e70772. doi: 10.1111/acel.70772 (PMC13696439; doi:10.1111/acel.70772)
Supplement: Supplementary file 2 [file ACEL-25-e70772-s002.docx]

**Supplementary Methods**

**Table M1** Life history traits of the five species studied.

**Metabolomics Analysis**

Metabolite extraction and quantification were performed using a high-performance liquid chromatography system (HPLC 1260 Infinity II, Agilent Technologies, Santa Clara, CA, USA) coupled to a triple quadrupole mass spectrometer (6420B Triple Quad, Agilent Technologies). The extracts were separated on an InfinityLab Poroshell 120 HILIC-Z column (2.7 μm, 10 × 2.1 mm). Two acquisition modes were applied, involving two mobile phases and two chromatographic separation methods, in both positive and negative ionization modes. The HPLC and mass spectrometer parameters for each detection mode are provided below.

Precursor ion, product ion, RT and QQQ parameters for each compound in positive mode:

Precursor ion, product ion, RT and QQQ parameters for each compound in negative mode:

**Statistical Analysis**

**Figure M1.** *Relationship between alanine concentration and ordinal species classification from the ordinal logistic regression model in mantle.* **A**: Predicted probabilities of belonging to each species (colored curves) as a function of scaled alanine concentration, obtained from the fitted ordinal logistic regression. Vertical dashed lines indicate the estimated thresholds on the predictor scale ($X_{threshold}= \zeta_{j}/\beta_{1}$), which correspond to the concentration values where the predicted probability of being in one category versus the next would reach 50%. The horizontal red dashed line highlights this 50% probability level, making explicit the link between threshold positions and the probability curves. Note that for some categories whose predicted probability never exceeds 50%, the model still estimates thresholds based on the regression parameters; in these cases, the threshold reflects the model’s theoretical cutpoint, even if it does not correspond to a point where the predicted probabilities visibly cross. Overlaid colored jittered points represent individual sample concentrations, showing the distribution of the predictor across categories. **B**: Linear predictor η(x) as a function of scaled alanine concentration, computed as η(x) = $\beta_{1}$·x, where $\beta_{1}$ is the estimated regression coefficient (for quadratic model, η(x) = $\beta_{1}$·x, + $\beta_{2}$·x²). Horizontal dashed lines correspond to the estimated thresholds $\zeta_{j}$, which partition the range of η(x) into intervals associated with each ordinal category. Values of η(x) below a given threshold indicate a higher probability of being in a lower longevity category, while values above a threshold indicate a higher probability of being in a higher longevity category. The zero line marks the point where the log-odds of belonging to a given category are balanced, corresponding to the steepest change in predicted probabilities**.** Because the model satisfies the Brant postulate, the slope of η(x) with respect to alanine concentration is constant across all thresholds, indicating that alanine has the same relative effect regardless of the position of the threshold separating the species categories.

**References**

Bourdages H & Goudreau P (2012) Évaluation de la pêche à la mactre de Stimpson (*Mactromeris polynyma*) des eaux côtières du Québec en 2011. *Secrétariat canadien de consultation scientifique du MPO, Document de recherche* 2012/008, 101 p.

Brulotte S & Giguère M (2007) Reproduction et taille à la maturité sexuelle de la mye commune (*Mya arenaria*) au Québec. *Rapport technique canadien des sciences halieutiques et aquatiques* 2698, viii + 40 p.

Butler PG, Wanamaker AD Jr, Scourse JD, Richardson CA & Reynolds DJ (2013) Variability of marine climate on the North Icelandic Shelf in a 1357-year proxy archive based on growth increments in the bivalve *Arctica islandica*. *Palaeogeography, Palaeoclimatology, Palaeoecology* 373, 141–151. https://doi.org/10.1016/j.palaeo.2012.01.016

Giguère M, Brulotte S, Paille N & Fortin J (2005) Mise à jour des connaissances sur la biologie et l’exploitation de la mactre de l’Atlantique (*Spisula solidissima*) aux Îles-de-la-Madeleine. *Rapport technique canadien des sciences halieutiques et aquatiques* 2587, 32 p.

Kilada RW, Campana SE & Roddick D (2007) Validated age, growth, and mortality estimates of the ocean quahog (*Arctica islandica*) in the western Atlantic. *ICES Journal of Marine Science* 64, 31–38. <https://doi.org/10.1093/icesjms/fsl001>

Lambert J & Goudreau P (1997) Biologie et exploitation de la mactre de Stimpson (*Mactromeris polynyma*) sur les côtes du Québec. *Secrétariat canadien pour l’évaluation des stocks du MPO, Document de recherche* 1997/101, 44 p.

Landry T & Sephton TW (1996) Southern Gulf Northern quahaug. *Fisheries and Oceans Canada Atlantic Fisheries Stock Status Report* 96/102E, 3 p.

MacDonald BA & Thomas MLH (1980) Age determination of the soft-shell clam *Mya arenaria* using shell internal growth lines. *Marine Biology* 58, 105–109. https://doi.org/10.1007/BF00396121

Munro D & Blier PU (2012) The extreme longevity of *Arctica islandica* is associated with increased peroxidation resistance in mitochondrial membranes. *Aging Cell* 11, 845–855. <https://doi.org/10.1111/j.1474-9726.2012.00847.x>

Ridgway ID, Richardson CA, Enos E, Ungvari Z, Austad SN, Philipp EER & Csiszar A (2011) New species longevity record for the Northern quahog (= hard clam), *Mercenaria mercenaria*. *Journal of Shellfish Research* 30, 35–38. [https://doi.org/10.2983/035.030.0106](https://doi.org/10.2983/035.030.0106?utm_source=chatgpt.com)

Roddick D, Mombourquette K & Kilada R (2007) 2002 survey for ocean quahogs (*Arctica islandica*) at the mouth of St. Marys Bay, Nova Scotia. *Canadian Science Advisory Secretariat Research Document* 2007/037, 36 p.

Sephton TW & Bryan CF (1990) Age and growth rate determinations for the Atlantic surfclam, *Spisula solidissima* (Dillwyn 1817), in Prince Edward Island, Canada. *Journal of Shellfish Research* 9, 177–186.
